# Pulmonary artery pseudoaneurysm-induced massive hemoptysis after chemotherapy combined with tislelizumab for lung squamous cell carcinoma: a case report

**DOI:** 10.3389/fmed.2025.1524248

**Published:** 2025-04-03

**Authors:** Xue-Jiao Yang, Yong-Juan Wu, Jing-Zhong Wang, Yu-Lan Zheng, Xiao-Qi Li

**Affiliations:** ^1^Department of Respiratory and Critical Care Medicine, Xiangyang Central Hospital, Affiliated Hospital of Hubei University of Arts and Science, Xiangyang, China; ^2^Department of Interventional Radiology, Xiangyang Central Hospital, Affiliated Hospital of Hubei University of Arts and Science, Xiangyang, China

**Keywords:** lung squamous cell carcinoma, massive hemoptysis, pulmonary artery pseudoaneurysm, tislelizumab, tumor necrosis

## Abstract

Lung squamous cell carcinoma (SCC) is a subtype of non-small cell lung cancer with high incidence and mortality rates. While chemotherapy and immune checkpoint inhibitors (ICIs) have become crucial treatment options for SCC, these may be associated with unforeseen complications. Here, we present a 65-year-old male of hemoptysis caused by a pulmonary artery pseudoaneurysm (PAP) after chemotherapy combined with tislelizumab for lung SCC. After confirmation with angiography, successful embolization was performed to occlude the pseudoaneurysm and proximal artery. To our knowledge, this is the first reported case of PAP formation after chemotherapy combined with tislelizumab for lung SCC.

## Highlights

• After undergoing anti-tumor treatment for pulmonary squamous cell carcinoma, the occurrence of hemoptysis warrants vigilance for pulmonary artery pseudoaneurysm (PAP).
• Tumor necrosis leading to cavitation may be a contributing factor in the formation of a PAP.
• CT angiography can confirm the diagnosis of PAP.
• Endovascular embolization is an effective treatment method.

## 1 Introduction

With advancements in medical technology, chemotherapy and immune checkpoint inhibitors (ICIs) have become crucial treatment options for lung squamous cell carcinoma (SCC) (1). Tislelizumab is a modified antibody targeting programmed death protein-1 (PD-1) that was approved for treating non-small cell lung cancer (NSCLC) in combination with chemotherapy (2). Tislelizumab can specifically bind to the PD-1 on T cell surface, effectively blocking its interaction with programmed death-ligand 1 (PD-L1) expressed on tumor cells (2). By preventing this binding, tislelizumab reverses PD-1-mediated immunosuppression, restores T cell activation, and enhances anti-tumor immune responses. However, combination therapy may be potentially associated with unforeseen complications that severely affect patient prognosis. This report describes a case of massive hemoptysis caused by a pulmonary artery pseudoaneurysm (PAP) after chemotherapy combined with tislelizumab for lung SCC, providing a reference for clinical practitioners.

## 2 Case report

**FIGURE 1:**
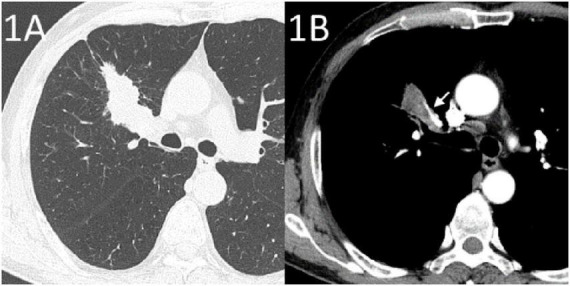
Enhanced CT at the diagnosis of lung cancer. **(A)** Lung window showing a tumor in the right upper lung. **(B)** Mediastinal window showing the margin of the tumor in the right upper lung in relation to the pulmonary artery (arrow).

**FIGURE 2:**
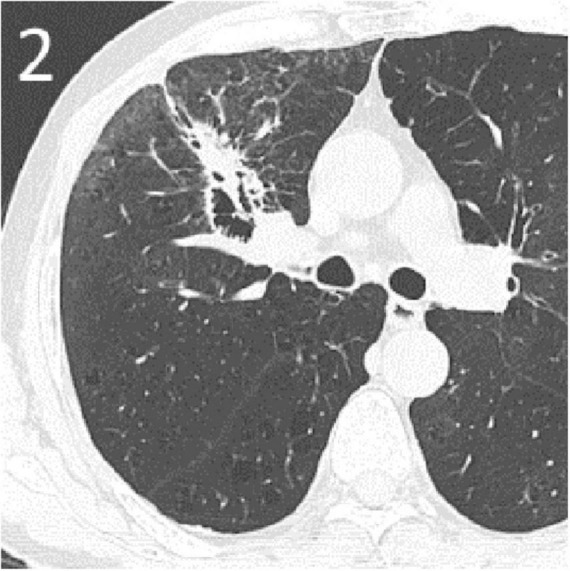
Re-examination plain CT after one cycle of chemotherapy combined with tislelizumab treatment. Lung window showing extensive necrosis of the tumor in the right upper lung with cavity formation.

A 65-year-old male was admitted to our hospital with sudden massive hemoptysis. The patient denied experiencing chest pain, dyspnea, or fever. He had a 20-year history of chronic obstructive pulmonary disease, but no other significant comorbidities such as vascular diseases, and was not receiving any other chronic therapies at the time of presentation. Two months prior, an enhanced CT scan suggested a tumor in the right upper lung (Figure 1), and a biopsy confirmed right upper lung SCC (TNM stage T2bNXM1c1). The patient received two cycles of albumin-bound paclitaxel, nedaplatin, and tislelizumab. A follow-up CT scan performed showed partial remission of the tumor with cavitation (Figure 2).

Upon physical examination, the patient’s hemodynamics were stable, and he exhibited no dyspnea but had hemoptysis with an oxygen saturation of 93%, a blood pressure of 132/67 mmHg, and a heart rate of 88 bpm. Approximately 200 mL of blood loss was estimated based on clinical observation. Auscultation revealed a few wet rales in the upper right lung. Laboratory tests showed hemoglobin at 78 g/L (normal range 130–175 g/L), white blood cell count at 13.46 x 10^9/L (normal range 3.50–9.50 x 10^9/L), neutrophil percentage at 87.9% (normal range 40–75.0%), and C-reactive protein at 143.0 mg/L (normal range 0–10 mg/L). Coagulation tests indicated a slightly prolonged prothrombin time (PT) of 13.5 s (normal range 9.0–13.0 s), a normal thrombin time (TT) of 15.7 s (normal range 14.0–21.0 s), an international normalized ratio (INR) of 1.17 (normal range 0.76–1.24), a PT activity of 67.6% (normal range 70.0–150.0%), and an activated partial thromboplastin time (APTT) of 29.4 s (normal range 21.5–37.0 s). Plasma fibrinogen was elevated at 6.15 g/L (normal range 2.00–4.00 g/L), and D-dimer was at the upper normal limit of 0.55 mg/L (normal range 0.00–0.55 mg/L).

**FIGURE 3:**
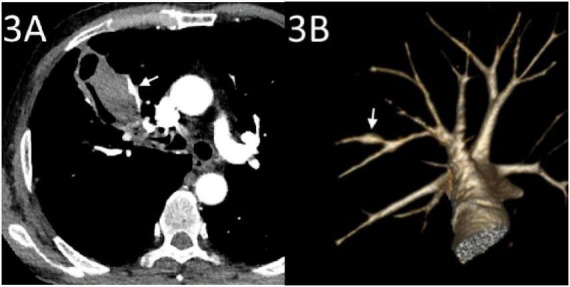
CT angiography (CTA) conducted after hemoptysis. **(A)** CTA showing a pseudoaneurysm of the pulmonary artery (arrow) at the edge of the cavity in the right upper lung tumor with hematoma inside the cavity. **(B)** Volume rendering reconstruction showing a pseudoaneurysm in a small branch of the left upper pulmonary artery (arrow).

**FIGURE 4:**
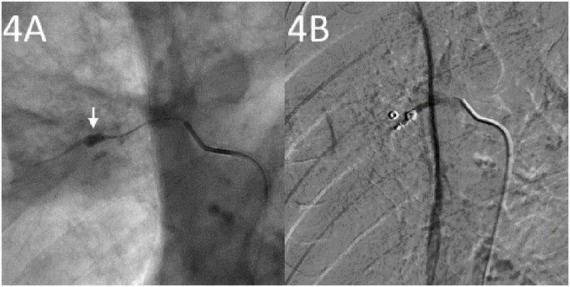
Angiography studies. **(A)** Super selective insertion of a microcatheter into the involved pulmonary artery showing a pulmonary artery pseudoaneurysm (arrow). **(B)** Post-embolization with coils showing pulmonary artery pseudoaneurysm success embolization.

Emergency CTA revealed pseudoaneurysm formation in the right upper pulmonary artery located at the SCC cavity wall with an intraluminal hematoma (Figure 3). The bronchial and non-bronchial systemic arteries showed no significant dilation. Emergency endovascular intervention was performed: after angiography confirmed the presence of a pseudoaneurysm, a 5F sheath (Outlook, Terumo, Tokyo, Japan) was inserted via the right femoral vein. Using a guidewire exchange, a 5F single-curve catheter (Cordis Corp., Miami Lakes, FL, USA) was advanced to the opening of the right upper pulmonary artery. A 2.1F microcatheter (Soft; Asahi Intecc Co., Ltd., Seto, Japan) was then super selectively inserted into the PAP. After confirmation via angiography, embolization was performed using microcoils (one with 4-mm diameter and 14-cm length, two with 3-mm diameter and 3-cm length; Cook, Bloomington, Ind) to occlude the PAP and proximal artery (Figure 4A). Post-procedure angiography confirmed the disappearance of the PAP (Figure 4B).

**FIGURE 5:**
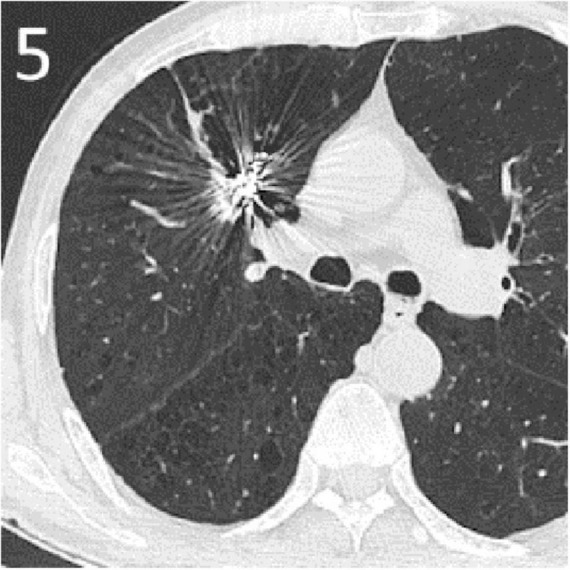
A CT scan 3 months post-embolization showed absorption of the tumor cavity with significant tumor reduction.

The patient experienced no active bleeding postoperatively, and follow-up over 6 months showed no recurrence of hemoptysis. Computed tomography (CT) scan 3 months post-embolization showed absorption of the tumor cavity with significant tumor reduction (Figure 5). Informed consent has been obtained for this report.

## 3 Discussion

Pulmonary artery pseudoaneurysm is a rare but extremely dangerous complication encountered in clinical practice (3, 4). Its formation mechanisms may include trauma, infection, tumor invasion, and iatrogenic factors (5). Primary lung cancer or pulmonary metastases can lead to PAP, a rare phenomenon involving direct tumor invasion and vascular wall erosion (4). SCC is the most common tumor because of its biological propensity for necrosis, and cavity formation is an independent risk factor for severe hemoptysis (4, 6). Additionally, studies have indicated that radiotherapy and chemotherapy can promote PAP formation by damaging the tumor and vascular walls (4, 6). ICIs enhance the immune response and significantly impact tumor cell death, leading to severe complications such as diffuse alveolar hemorrhage (7). While no reports have previously linked ICIs to PAP formation, a recent case report described fatal hemoptysis following treatment with tislelizumab and anlotinib in a patient with pulmonary sarcomatoid carcinoma, suggesting a potential association between ICI-based combination therapy and an increased risk of bleeding (8). Meanwhile, acquired PAP has been identified as a rare complication of systemic chemotherapy, as reported in another case report (9); unfortunately, no causal relationship was confirmed. In our case, the patient had no recent trauma, pulmonary infection, or history of receiving other invasive procedures. Additionally, he was not undergoing any other chronic treatments that could have compromised vascular integrity and did not show coagulation disorders according to laboratory tests. Therefore, the formation of PAP may result from dual damage to the pulmonary artery wall by SCC invasion and combined chemotherapy with tislelizumab, with exposed pulmonary arterial regions in the tumor necrotic cavity lacking tissue coverage, leading to pseudoaneurysm formation and rupture. Targeted treatment was immediately initiated upon diagnosis. Endovascular embolization was performed using interventional radiology to treat the pseudoaneurysms successfully (5, 10).

Reflecting on the clinical management of this case, early identification of high-risk factors, such as rapid tumor necrosis and cavitation, and close monitoring with periodic computed tomography angiography (CTA) might have facilitated earlier detection and potentially prevented severe hemoptysis (11). Despite successful embolization, closer monitoring during the initial cycles of combined therapy could have helped identify the early signs of vascular complications. Given the favorable outcome achieved by prompt embolization, we advocate immediate interdisciplinary consultation with interventional radiology or vascular surgery when PAP is suspected. In particular, patients presenting moderate-to-massive hemoptysis require continuous monitoring of vital signs and prompt initiation of emergency endovascular embolization, and potentially escalated care. Patients experiencing respiratory failure, hemorrhagic shock, or other severe complications should be immediately transferred to an intensive care unit for advanced supportive management. However, it remains uncertain whether routine advanced imaging would significantly improve outcomes, highlighting the need for further studies.

There are a few limitations to this case report. Firstly, while we hypothesize that the combination of tislelizumab and chemotherapy contributed to PAP formation through tumor necrosis and immune-mediated vascular injury, there is no direct evidence establishing a causal relationship. PD-L1 testing was also not performed prior to initiating chemotherapy and tislelizumab therapy for this patient because PD-L1 expression analysis was not available at our hospital at the time of treatment. Due to the lack of relevant reports and mechanistic studies, it remains unclear whether tislelizumab played a critical role in this case. Secondly, this is a single-case report, limiting the generalizability of our findings. Larger case series and retrospective studies are required to evaluate the potential risk associated with tislelizumab, or ICI-based combination therapy. Despite these limitations, this case highlights the need for vigilance regarding rare but severe complications, such as PAP, in the treatment of lung SCC with chemotherapy and tislelizumab. Cavitation within tumors may be an important factor in the formation of PAPs. Endovascular embolization is a safe and effective treatment method.

## Data Availability

The original contributions presented in this study are included in this article/supplementary material, further inquiries can be directed to the corresponding author.
